# Correction: fMRI reveals neural activity overlap between adult and infant pain

**DOI:** 10.7554/eLife.08663

**Published:** 2015-05-28

**Authors:** Sezgi Goksan, Caroline Hartley, Faith Emery, Naomi Cockrill, Ravi Poorun, Fiona Moultrie, Richard Rogers, Jon Campbell, Michael Sanders, Eleri Adams, Stuart Clare, Mark Jenkinson, Irene Tracey, Rebeccah Slater

Goksan S, Hartley C, Emery F, Cockrill N, Poorun R, Moultrie F, Rogers R, Campbell J, Sanders M, Adams E, Clare S, Jenkinson M, Tracey I, Slater R. 2015. fMRI reveals neural activity overlap between adult and infant pain. *eLife* **4**:e06356. doi: 10.7554/eLife.06356. Published 21 April 2015

Table 1 states that MNI/Neonate template coordinates are reported whereas they are voxel coordinates that incorrectly identify the peak voxels. This does not affect the results of the study and Table 1 has been updated to include the correct coordinates and anatomical nomenclature. For comparison, the originally published version of the table is available as Supplementary file 1 (file held on figshare under http://dx.doi.org/10.6084/m9.figshare.1418230).

Three referencing errors were also identified. In the Materials and methods (section Data analysis: MR Data) the Cornelissen et al., 2013 reference should have been the Serag et al., 2012 reference, and the Poldrack et al., 2008 reference should have been the Jenkinson et al., 2002 reference. Reference to Williams et al., 2015 was omitted. The Abstract and main text have been updated to reflect the inclusion of the omitted reference.

We apologise for these errors. The article has been corrected accordingly.

